# Risco de Desfechos Adversos à Saúde em Pacientes com Baixa Adesão ao Tratamento Medicamentoso Cardiovascular: Uma Revisão Sistemática

**DOI:** 10.36660/abc.20240469

**Published:** 2024-10-24

**Authors:** Marcus Vinícius Bolívar Malachias, Sergio Emanuel Kaiser, Denilson Campos de Albuquerque, Andrea Araújo Brandão, Andrei Carvalho Sposito, Lidia Zytysnky Moura, Lucélia Batista Neves Cunha Magalhães, Marco Antonio Mota-Gomes, Nadine Clausell, Paulo César Veiga Jardim, Wilson Nadruz, Bruno Monteiro Barros, Leonardo Castro Luna, Weimar Kunz Sebba Barroso

**Affiliations:** 1 Faculdade Ciências Médicas de Minas Gerais FELUMA Belo Horizonte MG Brasil Faculdade Ciências Médicas de Minas Gerais, Fundação Educacional Lucas Machado (FELUMA), Belo Horizonte, MG – Brasil; 2 Universidade do Estado do Rio de Janeiro Rio de Janeiro RJ Brasil Universidade do Estado do Rio de Janeiro, Rio de Janeiro, RJ – Brasil; 3 Instituto D’Or de Pesquisa e Ensino-IDOR Rio de Janeiro RJ Brasil Instituto D’Or de Pesquisa e Ensino-IDOR, Rio de Janeiro, RJ – Brasil; 4 Faculdade de Ciências Médicas Universidade Estadual de Campinas Campinas SP Brasil Faculdade de Ciências Médicas, Universidade Estadual de Campinas (UNICAMP), Campinas, SP – Brasil; 5 Pontifícia Universidade Católica de Curitiba Curitiba PR Brasil Pontifícia Universidade Católica de Curitiba, Curitiba, PR – Brasil; 6 Faculdade Zarns Salvador BA Brasil Faculdade Zarns, Salvador, BA – Brasil; 7 Centro de Pesquisas Clínicas Dr. Marco Mota Centro Universitário CESMAC Maceió AL Brasil Centro de Pesquisas Clínicas Dr. Marco Mota, Centro Universitário CESMAC, Maceió, AL – Brasil; 8 Hospital de Clínicas de Porto Alegre Porto Alegre RS Brasil Hospital de Clínicas de Porto Alegre, Porto Alegre, RS – Brasil; 9 Hospital do Coração de Goiás Goiânia GO Brasil Hospital do Coração de Goiás, Goiânia, GO – Brasil; 10 Unidade de Hipertensão Arterial Faculdade de Medicina Universidade Federal de Goiás Goiânia GO Brasil Unidade de Hipertensão Arterial, Faculdade de Medicina, Universidade Federal de Goiás, Goiânia, GO – Brasil

**Keywords:** Adesão à Medicação, Cooperação do Paciente, Doenças Cardiovasculares

## Abstract

**Fundamento:**

As doenças cardiovasculares (DCV) são a principal causa de mortes no mundo. A adesão ao tratamento medicamentoso é um fator importante no manejo de DCV crônicas, influenciando diretamente a ocorrência de desfechos e custos da saúde.

**Objetivos:**

Esta revisão sistemática, apoiada pela Sociedade Brasileira de Cardiologia, tem como objetivo avaliar o impacto da baixa adesão aos medicamentos para DCV sobre desfechos clínicos críticos como morte e eventos cardiovasculares.

**Métodos:**

Foi realizada uma busca abrangente em quatro bases de dados: Medline, Embase, Lilacs e Cochrane. Foram incluídas revisões sistemáticas e metanálises que avaliaram estimativas de risco para adesão aos medicamentos cardiovasculares. Quatro revisões sistemáticas, cada uma incorporando estudos observacionais, foram selecionadas.

**Resultados:**

O aumento na adesão aos medicamentos reduz significativamente o risco de eventos cardiovasculares, acidente vascular cerebral (AVC) e morte por todas as causas. Uma melhoria de 20% na adesão a medicamentos anti-hipertensivos, hipolipemiantes e outros medicamentos cardiovasculares foi correlacionada com reduções nos eventos cardiovasculares em 7%, 10% e 9%, respectivamente; AVC em 17%, 13% e 18%; e morte em 12%, 9% e 10%. A certeza das evidências foi moderada, sugerindo que esses efeitos provavelmente estão presentes. Esses achados enfatizam a necessidade de aumentar a adesão aos tratamentos para melhorar os desfechos clínicos no manejo das DCV.

**Conclusões:**

Evidências demonstraram reduções em morte e desfechos críticos, tanto na prevenção primária quanto na secundária, por meio do controle de condições como hipertensão e colesterol LDL elevado, assim como benefícios da terapia antiplaquetária na doença aterosclerótica. Mais estudos são necessários para melhor elucidar a relação entre a adesão aos medicamentos cardiovasculares e a melhora de desfechos clínicos críticos.

## Graphical abstract

**Figura Central:**
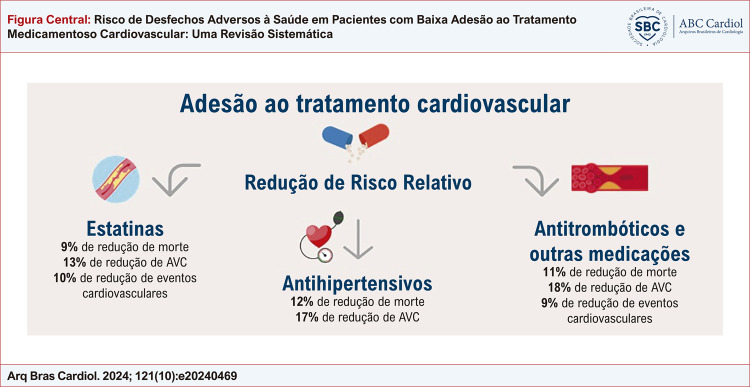
: Risco de Desfechos Adversos à Saúde em Pacientes com Baixa Adesão ao Tratamento Medicamentoso Cardiovascular: Uma Revisão Sistemática

**Figura Central:** : Risco de Desfechos Adversos à Saúde em Pacientes com Baixa Adesão ao Tratamento Medicamentoso Cardiovascular: Uma Revisão Sistemática

Adesão ao Tratamento Medicamentoso Cardiovascular.

## Introdução

As doenças cardiovasculares (DCV) continuam sendo um desafio importante para a saúde global; são a principal causa de mortalidade em todo o mundo, sendo responsáveis por estimadas 17,9 milhões de mortes anualmente, ou 31% dos óbitos totais.^1^ Fatores de risco como hipertfensão, hipercolesterolemia, diabetes, tabagismo, obesidade e estilo de vida sedentário, contribuem significativamente para a prevalência das DCV e para a ocorrência de mortes prematuras. A necessidade de implementar estratégias de saúde pública focadas em modificações de estilo de vida e cuidados preventivos é crucial.^2^

Do ponto de vista econômico, as DCV impõem uma carga significativa, não apenas pelos custos diretos com a saúde, mas também pelos custos indiretos associados à perda de produtividade e incapacidades de longo prazo.^3^

O objetivo desta revisão sistemática é, por meio da busca pelas melhores evidências científicas disponíveis, avaliar o impacto da adesão ao tratamento medicamentoso cardiovascular nos desfechos clínicos. Este documento servirá de base para um Posicionamento Clínico da Sociedade Brasileira de Cardiologia sobre o tema.

## Métodos

A pergunta de pesquisa, formulada no formato PICO (paciente/população, intervenção, comparação e desfechos), foi: na população adulta, com 18 anos ou mais, quais são as diferenças nos desfechos clínicos (morte, acidente vascular cerebral (AVC) e infarto do miocárdio) entre pacientes que aderem adequadamente ou não ao tratamento medicamentoso cardiovascular? O protocolo para este documento foi aprovado pelo patrocinador e está disponível para consulta mediante solicitação aos autores.

A metodologia utilizada neste documento a revisão sistemática rápida, pertencente à família das revisões sistemáticas. Trata-se de uma ferramenta desenvolvida na última década com o objetivo de manter o rigor metodológico ao buscar as melhores evidências possíveis, mas com modificações que agilizam o tempo de execução. Tipicamente, essas revisões informam sociedades médicas ou instituições de saúde sobre as melhores evidências disponíveis, baseadas em uma pergunta formatada em PICO de forma sensível, transparente e sistemática. Instituições líderes no campo da metodologia estabeleceram os métodos desse tipo de documento.^4-6^

Foi realizada uma busca abrangente em quatro bases de dados: Medline, Embase, Lilacs e Biblioteca Cochrane, incluindo todos os registros desde o início até 1º de março de 2024.

Dois pesquisadores conduziram independentemente a seleção dos estudos e a avaliação da qualidade das revisões sistemáticas por uma fase inicial em que ambos trabalharam juntos até alcançar uma taxa de concordância de 90%, após a qual cada documento poderia ser avaliado por um único autor. Para extração de dados, um pesquisador extraiu todas as variáveis predefinidas em uma planilha, enquanto o segundo pesquisador extraiu independentemente apenas os dados de efeito. A avaliação da qualidade das revisões sistemáticas foi realizada usando duas ferramentas específicas – AMSTAR 2 - *A Measurement Tool to Assess Systematic Reviews* e a Lista de Verificação de Revisão Sistemática do Instituto Joanna Briggs (JBI).^7,8^ Além disso, a estrutura GRADE foi empregada para avaliar a qualidade das evidências e determinar a força das recomendações, quando possível.^5^

Os artigos selecionados para leitura completa foram considerados para inclusão no estudo se atendessem aos seguintes critérios: (1) fossem revisões sistemáticas que incluíssem metanálises; (2) relatassem estimativas de risco para avaliar o impacto da adesão sobre eventos cardiovasculares importantes (morte por qualquer causa, AVC e infarto do miocárdio); (3) incluíssem pacientes com 18 anos ou mais; (4) avaliassem pelo menos um grupo de medicamentos cardiovasculares, como anti-hipertensivos, hipolipemiantes, antitrombóticos ou antiplaquetários.

Os seguintes critérios de exclusão foram aplicados: (1) revisões que utilizaram menos de duas bases de dados em sua busca; (2) revisões sem metanálises detalhadas; (3) revisões que não analisaram a qualidade metodológica dos estudos primários; (4) estudos com baixa qualidade de evidência, conforme avaliado por ambas as ferramentas (AMSTAR-2 e JBI). Não houve restrições de idioma na seleção dos estudos.

Os detalhes das estratégias de busca e da metodologia empregada nesta revisão sistemática rápida estão disponíveis no material suplementar (Tabela 1S).

## Resultados

Dos 643 registros identificados, 15 foram selecionados para leitura completa por preencherem os critérios de elegibilidade. Ao final, quatro revisões sistemáticas com metanálises foram incluídas (Figura 1S, material suplementar). Uma lista de estudos excluídos e os motivos para suas exclusões estão apresentados no material suplementar (Tabela 2S).

As quatro revisões sistemáticas incluíram apenas estudos observacionais, conforme o esperado. Uma das revisões avaliou a adesão a vários tipos de medicamentos cardiovasculares e uma gama mais ampla de desfechos, além de medir a curva dose-resposta entre a adesão e as complicações.^9^ Esta revisão é considerada a melhor evidência disponível e serviu de base para as conclusões deste artigo. As outras três revisões focaram exclusivamente em um grupo de medicamentos: anti-hipertensivos,^10^ estatinas^11^ ou ácido acetilsalicílico.^12^ A Tabela 1 apresenta as principais características avaliadas nos estudos incluídos.

**Tabela 1:** – Características dos estudos incluídos na revisão rápida

Autor/ Ano	Nº de estudos incluídos	Tipo de estudos	Medicamento	Desfechos	População	Medida de adesão	Participantes (n)	Idade mediana	Mulheres (%)	Financiamento
Liu et al. 2021^9^	46	Observacional	Estatinas, anti-hipertensivos, antidiabéticos e antitrombóticos	Eventos cardiovasculares, AVC e mortalidade por todas as causas	Hipertensão / Dislipidemia	TPM, PMC, ACM, PDC	4 051 338	60,1	44	Não
Lee et al. 2022^10^	161	Obser-vacional	Anti-hipertensivos	Morte e hospitalização	Pacientes com hipertensão, >18 anos; excluídos: hipertensão resistente em gestantes	MMAS de 4 ou 8 itens, contagem de comprimidos, recarga de receitas, caixas de comprimidos eletrônicas, ensaios bioquímicos ou monitoramento eletrônico de medicamentos	27 785 595	57	57,1	Não
Xu et al. 2016^11^	6	Obser-vacional	Estatina	Mortalidade por todas as causas, recorrência de DCV e revascularização	DCV	PDC	38 301	NR	NR	Não
Biondi-Zoccai et al. 2006^12^	6	Obser-vacional	Aspirina	Eventos cardiovasculares	Pacientes em risco ou com DAC	NR	50 279	NR	NR	NR

ACM: adesão cumulativa ao medicamento; AVC: acidente vascular cerebral; DAC: doença arterial coronariana; DCV: doença cardiovascular; MMAS: Escala de Adesão Medicamentosa de Morisky; NR: não relatado; PDC: Proporção de Dias Cobertos; PMC: proporção de meses cobertos por prescrição; TPM: taxa de posse de medicação.

Fonte: Autores.

Liu et al.^9^ avaliaram a associação entre a adesão a medicamentos cardiológicos e o risco de eventos cardiovasculares, AVC e mortalidade por todas as causas. Os estudos incluíram pacientes tanto em prevenção primária quanto secundária, envolvendo indivíduos saudáveis, e indivíduos com hipertensão, diabetes, dislipidemia, e DCV preexistentes. Os medicamentos avaliados foram hipolipemiante, anti-hipertensivos, antidiabéticos e agentes antitrombóticos. Os desfechos avaliados foram morte por todas as causas, AVC e eventos cardiovasculares (definidos como qualquer doença coronariana fatal ou não fatal, infarto do miocárdio, insuficiência cardíaca, doença isquêmica do coração ou morte súbita cardíaca). As estimativas de risco relativo (RR) específicas de cada estudo foram calculadas para cada incremento de 20% na adesão ao medicamento e, em seguida, agrupadas. Mais de quatro milhões de pacientes, distribuídos em 46 estudos observacionais, foram incluídos na análise, com uma avaliação de qualidade usando a Escala Newcastle–Ottawa, obtendo uma pontuação média de 7,9 (máximo de 9), e um período médio de acompanhamento de 4,6 anos. A análise mostrou que o aumento de 20% na adesão a medicamentos anti-hipertensivos, hipolipemiantes e outros medicamentos cardiovasculares reduziu o risco de eventos cardiovasculares em 7% [RR 0,93 (IC 95%, 0,84-1,03), não significativo], com outros achados demonstrando significância: 10% [RR 0,90 (0,88-0,92)] e 9% [RR 0,91 (0,84-0,98)], respectivamente. Esse aumento na adesão também reduziu o risco de AVC em 17% [RR 0,83 (0,78-0,89)], 13% [RR 0,90 (0,88-0,92)] e 18% [RR 0,91 (0,84-0,98)], além de reduzir a mortalidade por todas as causas em 12% [RR 0,88 (0,82-0,94)], 9% [RR 0,91 (0,89-0,94)] e 10% [RR 0,89 (0,84-0,94)], respectivamente (Figura Central). Uma análise de sensibilidade e subgrupos realizada para os vários desfechos não mostrou diferença significativa nas estimativas agrupadas associadas à boa adesão ao tratamento medicamentoso cardiovascular.

Lee et al.^10^ conduziram uma revisão sistemática e metanálise para estimar a prevalência global e as consequências da não adesão a medicamentos anti-hipertensivos em pacientes adultos hipertensos. A análise incluiu vários métodos de medição da adesão ao tratamento e envolveu 161 estudos observacionais. Essa metanálise de prevalência teve como objetivo principal avaliar o controle da pressão arterial, além de estimar desfechos secundários, como complicações relacionadas à hipertensão, hospitalização por todas as causas e mortalidade por todas as causas. Os resultados indicaram que a não adesão aos medicamentos anti-hipertensivos estava associada a um aumento no odds ratio (OR) de morte de 1,38 [IC 95%, 1,35-1,41]. No entanto, esses resultados foram baseados em apenas dois estudos com 1.653.763 pacientes e um seguimento mediano de 4,5 anos. A análise da certeza do corpo de evidências, utilizando a análise GRADE, foi considerada baixa para todos os desfechos. Esses resultados devem-se principalmente à natureza observacional dos estudos.^5^

Xu et al.^11^ avaliaram, por meio de uma metanálise, a relação entre a adesão ao uso de estatinas e as consequências clínicas de longo prazo em pacientes com DCV (prevenção secundária). O desfecho primário foi a mortalidade por todas as causas, medida pelo RR. O método de proporção de dias cobertos (PDC) foi utilizado para quantificar a adesão às estatinas (PDC ≥80% - boa adesão e PDC <80% - baixa adesão). Um total de seis estudos foi incluído nessa metanálise, e o RR combinado favorável à boa adesão às estatinas foi de 0,64 (indicando uma redução de 36% no risco de morte por qualquer causa) [IC 95%, 0,52-0,80]. Houve significância estatística, embora o intervalo de confiança amplo indicasse uma imprecisão notável.

A última metanálise incluída foi a de Biondi-Zoccai et al.,^12^ em que os perigos inerentes à retirada ou não adesão ao ácido acetilsalicílico em indivíduos com risco de ou com doença arterial coronariana foram avaliados. Seis estudos foram selecionados, e o resultado da estimativa agrupada revelou que a não adesão ou retirada do antiplaquetário estava associada a um risco três vezes maior de eventos cardíacos adversos graves.

A Tabela 2 fornece o RR dos desfechos avaliados e o número de estudos incluídos em cada estimativa resumida.

**Tabela 2:** – Resultados das metanálises dos estudos incluídos

Autor principal / Ano	Grupo de medicamentos	Mortalidade por todas as causas Medida (IC 95%)	Nº de estudos	AVC Medida (IC 95%)	Nº de estudos	Eventos cardiovasculares Medida (IC 95%)	Nº de estudos	Efeito dose-resposta
Liu et al. 2021^9^	Qualquer medicamento cardiovascular*	RR 0,90 (0,87-0,92)	26	RR 0,84 (0,81-0,87)	23	RR 0,91 (0,88-0,94)	35	Yes
	Agentes redutores de lipídios*	RR 0,91 (0,89-0,94)	12	RR 0,87 (0,84-0,91)	7	RR 0,90 (0,88-0,92)	17	Yes
	Anti-hipertensivos*	RR 0,88 (0,82-0,94)	8	RR 0,83 (0,78-0,89)	12	RR 0,93 (0,84-1,03)	13	Yes
	Outros*†	RR 0,89 (0,84-0,94)	6	RR 0,82 (0,74-0,92)	4	RR 0,91 (0,84-0,98)	5	Yes
Lee et al. 2022^10^	Anti-hipertensivos	RR 0,75 (0,73-0,76) ‡	2	NR	-	NR	-	Não
Xu et al. 2016^11^	Estatinas	RR 0,64 (0,52-0,80)	6	NR	-	NR	-	Não
Biondi-Zoccai et al. 2006^12^	Aspirina	NR	-	NR	-	RR 0,37 (0,24-0,60) §	6	Não

(*) RR calculado para cada aumento de 20% na adesão ao medicamento. (†) Outros: agentes antitrombóticos e múltiplos medicamentos. (‡) RR invertido aproximado, calculado a partir do resultado de Odds Ratio original (1.38 [CI 1.35-1.41]) e uma probabilidade ponderada de 8.01% de eventos ao longo do seguimento. (§) RR invertido aproximado, calculado a partir do resultado de Odds Ratio original (3.14 [1.75-5.61]) e uma probabilidade ponderada de 7,5% de eventos ao longo do seguimento. NR: não relatado; OR: razão de chances; RR: risco relativo. O nível de significância estatística adotado em todos os estudos foi de 5%.

Fonte: Autores.

Os resultados da análise AMSTAR-2 e da lista de verificação de avaliação crítica do JBI estão apresentados nas Tabelas 3S e 4S (material suplementar). Apesar das diferenças nos resultados dessas duas ferramentas, os autores consideraram a qualidade geral das quatro revisões sistemáticas incluídas como satisfatória.

Os autores realizaram uma avaliação separada do GRADE para o estudo de Liu et al.^9^ com base no risco de viés e nas características extraídas do estudo. Essa avaliação abordou todos os grupos de medicamentos e os vários desfechos. Em quase todas as situações, as evidências alcançaram um nível moderado de certeza, em grande parte devido à presença de um gradiente dose-resposta. No entanto, a certeza das evidências para os desfechos de eventos cardiovasculares associados a medicamentos anti-hipertensivos foi considerada muito baixa. Essa avaliação foi baseada na imprecisão das estimativas de RR, que cruzaram a linha de efeito nulo. Consequentemente, foi recomendado não aumentar a pontuação de certeza para esses desfechos específicos.

A tabela de resumo das evidências está disponível no material suplementar (Tabela 5S).

## Discussão

A adesão ao tratamento medicamentoso é uma questão de saúde importante nos dias de hoje e a Sociedade Brasileira de Cardiologia adotou uma conduta pioneira ao abordar este tema, buscando as melhores evidências científicas para embasar seu posicionamento.

Responder as perguntas levantadas para esta revisão não é uma tarefa simples. Era esperado que não houvesse ensaios clínicos sobre o tema, uma vez que não é viável randomizar pacientes para aderirem ou não aos medicamentos cardiovasculares com benefícios comprovados. Portanto, foi necessário utilizar revisões sistemáticas de estudos observacionais que, embora comecem com um baixo grau de certeza das evidências, tentam agregar os resultados de vários estudos para melhorar a confiança na estimativa do efeito.

Além disso, era previsto que esses estudos sobre adesão apresentassem uma heterogeneidade significativa por diversos motivos: a grande variedade de métodos para medir a adesão, os diferentes grupos de medicamentos cardiovasculares, as populações diversas (prevenção primária vs. secundária, adultos vs. idosos, etc.), a pluralidade de desfechos clínicos e a qualidade variável dos estudos, entre outras possibilidades.

A adesão ao tratamento medicamentoso é frequentemente quantificada utilizando vários pontos de corte e categorizada em níveis distintos, fornecendo informações essenciais sobre o comportamento do paciente e a eficácia do tratamento. Geralmente, a adesão é classificada com base em limites percentuais que refletem a proporção de doses prescritas que um paciente toma durante um período específico. Comumente, “alta adesão” é definida como o consumo de 80% ou mais das doses prescritas, “adesão média” como o consumo de 50-79% e “baixa adesão” como o consumo de menos de 50%. Esses limites são amplamente utilizados na pesquisa clínica para avaliar a eficácia de intervenções e são cruciais para entender seu impacto nos desfechos clínicos.^13^ O estudo de referência de Liu et al.^9^ avaliou o impacto de um aumento de 20% na adesão medicamentosa (por exemplo, de 80% - o ponto de corte mais utilizado na literatura para distinguir boa adesão - para 100%). O estudo demonstrou uma diminuição significativa nos principais desfechos cardiovasculares quando os medicamentos cardiovasculares foram utilizados conforme prescrito.

Uma metanálise que examinou a terapia com estatinas revelou que as taxas de adesão após um ano de acompanhamento diferiram significativamente entre os tipos de estudo: 49,0% em estudos observacionais em comparação com 90,3% em ensaios clínicos randomizados (RCTs). Essa discrepância sugere que a adesão em RCTs, em que há maior controle, pode ser superestimada em comparação com a realidade prática.^14^

Esses pontos de corte de adesão são utilizados em diversos tipos de medicamentos, incluindo terapias crônicas e agudas, para padronizar as metodologias de pesquisa e permitir comparações significativas. Por exemplo, a Organização Mundial da Saúde indica que níveis de adesão acima de 80% são geralmente necessários para alcançar resultados terapêuticos ideais na maioria das condições crônicas.^15^ No entanto, certas condições exigem taxas de adesão mais específicas; por exemplo, a terapia antirretroviral para o HIV pode exigir níveis de adesão de até 95% para suprimir efetivamente a carga viral. Embora esses limites sejam essenciais para a uniformidade da pesquisa, sua aplicabilidade pode variar com base na janela terapêutica do medicamento, na condição de saúde específica e nos fatores individuais do paciente. Essa variabilidade ressalta a necessidade de estratégias de adesão adaptadas para maximizar os resultados dos pacientes, sugerindo uma aplicação mais individualizada desses critérios, dependendo dos requisitos terapêuticos e das circunstâncias do paciente.^16^

Estabelecer as barreiras multifacetadas à adesão é essencial para otimizar os desfechos cardiovasculares. Fatores relacionados aos pacientes (esquecimento, crenças sobre a medicação, efeitos colaterais percebidos), desafios socioeconômicos (custo dos medicamentos, educação do paciente) e obstáculos do próprio sistema de saúde (regimes de medicação complexos, falta de acompanhamento) devem ser abordados sistematicamente para melhorar as taxas de adesão.^15,17^ Diferentes modelos e teorias tentam abordar as complexidades envolvidas nesses comportamentos. Por exemplo, o Modelo de Crenças em Saúde sugere que as percepções dos pacientes sobre a gravidade de sua condição, os potenciais benefícios do tratamento e as barreiras ao cuidado podem influenciar a adesão e a persistência.^18^ Além disso, a Organização Mundial da Saúde identifica alguns fatores que impactam a adesão, categorizando-os em fatores relacionados ao paciente, à doença, à terapia, ao contexto socioeconômico e ao sistema de saúde.^15^ Educação do paciente, regimes de medicamentos simplificados e uma comunicação aprimorada entre prestadores de saúde e pacientes são essenciais para melhorar a adesão e, consequentemente, os desfechos clínicos no manejo das DCV.^17^

Um aspecto que também merece consideração nos estudos sobre adesão medicamentosa é a intensidade da dosagem. Por exemplo, uma investigação avaliou a adesão entre diferentes níveis de intensidade de estatinas — baixa, moderada e alta. Os resultados indicaram que as taxas de adesão em 12 meses foram de 57,2% para estatinas de baixa intensidade, 46,5% para estatinas de intensidade moderada e 37,9% para estatinas de alta intensidade, com a adesão definida como a obtenção de pelo menos 80% de conformidade com a medicação.^19^ Outro estudo identificou uma disparidade estatisticamente significativa na adesão entre estatinas de baixa e alta dosagem, sugerindo que regimes envolvendo estatinas de alta intensidade estão associados a menor adesão em comparação com os regimes de intensidade mais baixa.^20^ Essa observação parece contradizer um artigo mais recente, que afirma que, após a introdução das novas diretrizes da American College of Cardiology/American Heart Association (ACC/AHA), uma proporção maior de pacientes com DCV aterosclerótica não apenas recebe prescrições de estatinas de alta intensidade, mas também são mais propensos a aderir ao regime de tratamento.^21^

Outra questão importante nesse contexto é o uso da terapia de combinação em dose fixa (FDC, do inglês *fixed-dose combination*), que surgiu como uma estratégia promissora para melhorar a adesão medicamentosa em pacientes com DCV. A terapia FDC simplifica o regime de tratamento ao combinar múltiplos medicamentos em um único comprimido, o que pode reduzir a carga de comprimidos e melhorar a adesão dos pacientes.^22^ Estudos demonstraram que a terapia FDC está associada a maiores taxas de adesão e melhores desfechos clínicos em comparação com regimes de múltiplos comprimidos. Por exemplo, um estudo constatou que pacientes em terapia FDC apresentaram uma taxa de adesão 24% maior e controle significativamente melhor da pressão arterial. Da mesma forma, uma metanálise relatou que a terapia FDC levou a uma redução de 26% no risco de não adesão e a um melhor manejo dos fatores de risco cardiovasculares.^23^ Esses achados indicam os potenciais benefícios da terapia FDC na otimização do tratamento cardiovascular e na melhora da adesão dos pacientes.

Esta revisão não está isenta de limitações. Primeiramente, foram selecionadas apenas revisões sistemáticas que incluíram estudos observacionais. Estudos observacionais, por sua natureza, têm uma maior margem de imprecisão, principalmente devido a fatores de confusão não medidos ou desconhecidos. Isso justifica a necessidade de estudos primários para identificar e ajustar os fatores de confusão considerados importantes, e de revisões sistemáticas para avaliar a qualidade dos estudos primários, idealmente incluindo apenas pesquisas de alta qualidade. Alguns dos documentos selecionados não atenderam a esses critérios, e foram excluídos da nossa revisão.

Em segundo lugar, dado o interesse da Sociedade Brasileira de Cardiologia em várias classes de medicamentos cardiovasculares e em desfechos clínicos críticos, bem como a significativa variabilidade nos métodos de mensuração da adesão, era esperada uma heterogeneidade substancial entre os estudos. Em relação ao documento de Liu et al.,^9^ decidiu-se não penalizar o estudo pela heterogeneidade, apesar das estatísticas Cochran Q e I^2^ haverem indicado variabilidade significativa entre os estudos. O estudo apresentou estimativas de efeito semelhantes com uma direção consistente ao analisar os diferentes grupos de medicamentos por desfecho. Além disso, os intervalos de confiança demonstraram sobreposição, e várias análises de subgrupos não alteraram os resultados gerais, tampouco a análise de sensibilidade. Embora a heterogeneidade tenha sido apenas parcialmente explicada, os potenciais benefícios da boa adesão aos medicamentos cardiovasculares eficazes são substanciais e não devem ser subestimados. A decisão de não penalizar a avaliação do GRADE em relação a essa característica pode não ser unânime.

Por fim, em relação à avaliação da certeza das evidências utilizando a ferramenta GRADE, idealmente, essa avaliação deveria ser conduzida pelos autores em cada revisão sistemática com metanálise. No entanto, dos quatro estudos incluídos, apenas um apresentou tal análise. No estudo de referência, essa avaliação foi realizada com base apenas nas informações extraídas, sem acesso às fontes primárias.

Embora haja evidências robustas que apoiem a redução de desfechos duros tanto na prevenção primária quanto na secundária por meio do controle de variáveis clínicas como pressão arterial^24,25^e concentrações elevadas de LDL-colesterol,^26,27^ bem como os benefícios da terapia antiplaquetária na doença aterosclerótica,^28^ ainda há poucos estudos que demonstram correlações entre a adesão ao uso de medicamentos de ação cardiovascular e a atenuação de desfechos clínicos críticos.

Apesar das limitações inerentes aos estudos observacionais, as evidências dos riscos associados à baixa adesão medicamentosa reforçam a necessidade global de implementar estratégias que melhorem a adesão aos tratamentos cardiovasculares.

## Conclusão

Esta revisão sistemática demonstra o impacto significativo da boa adesão ao tratamento com medicamentos cardiovasculares nos desfechos clínicos.

De acordo com a metodologia GRADE, há uma certeza moderada das evidências de que pacientes que aderem adequadamente aos seus medicamentos cardiovasculares prescritos experimentam uma redução nas taxas de morte, AVC e desfechos cardiovasculares em comparação com indivíduos com menor adesão.
