# Supplementary material for: Risco de Desfechos Adversos à Saúde em Pacientes com Baixa Adesão ao Tratamento Medicamentoso Cardiovascular: Uma Revisão Sistemática
Source: Arq Bras Cardiol. 2024 Oct 24;121(10):e20240469. [Article in Portuguese] doi: 10.36660/abc.20240469 (PMC11634203; doi:10.36660/abc.20240469)
Supplement: Translation

# Translation: Risk of Adverse Health Outcomes in Patients with Poor Adherence to Cardiovascular Medication Treatment: A Systematic Review

**DOI:** 10.36660/abc.20240469i

**Authors:** Marcus Vinícius Bolívar Malachias, Sergio Emanuel Kaiser, Denilson Campos de Albuquerque, Andrea Araújo Brandão, Andrei Carvalho Sposito, Lidia Zytysnky Moura, Lucélia Batista Neves Cunha Magalhães, Marco Antonio Mota-Gomes, Nadine Clausell, Paulo César Veiga Jardim, Wilson Nadruz, Bruno Monteiro Barros, Leonardo Castro Luna, Weimar Kunz Sebba Barroso

**Affiliations:** 1 Brazil Faculdade Ciências Médicas de Minas Gerais, Fundação Educacional Lucas Machado (FELUMA), Belo Horizonte, MG – Brazil; 2 Brazil Universidade do Estado do Rio de Janeiro, Rio de Janeiro, RJ – Brazil; 3 Brazil Instituto D’Or de Pesquisa e Ensino-IDOR, Rio de Janeiro, RJ – Brazil; 4 Brazil Faculdade de Ciências Médicas, Universidade Estadual de Campinas (UNICAMP), Campinas, SP – Brazil; 5 Brazil Pontifícia Universidade Católica de Curitiba, Curitiba, PR – Brazil; 6 Brazil Faculdade Zarns, Salvador, BA – Brazil; 7 Brazil Centro de Pesquisas Clínicas Dr. Marco Mota, Centro Universitário CESMAC, Maceió, AL – Brazil; 8 Brazil Hospital de Clínicas de Porto Alegre, Porto Alegre, RS – Brazil; 9 Brazil Hospital do Coração de Goiás, Goiânia, GO – Brazil; 10 Brazil Unidade de Hipertensão Arterial, Faculdade de Medicina, Universidade Federal de Goiás, Goiânia, GO – Brazil

**Keywords:** Medication Adherence, Patient Compliance, Cardiovascular Diseases

## Abstract

**Background:**

Cardiovascular diseases (CVD) remain the leading cause of mortality worldwide. Medication adherence is an important issue in managing chronic CVD, directly influencing outcomes and healthcare costs.

**Objectives:**

This systematic review, supported by the Brazilian Society of Cardiology, evaluates the impact of poor adherence to cardiovascular medications on critical clinical outcomes such as death and cardiovascular events.

**Methods:**

A comprehensive search was conducted across four databases, including Medline, Embase, Lilacs, and the Cochrane Library. The review included systematic reviews with meta-analyses that reported risk estimates for adherence to cardiovascular medications. Four systematic reviews, each incorporating observational studies, were selected.

**Results:**

An increase in adherence to medications significantly reduces the risk of cardiovascular events, stroke, and all-cause death. Specifically, a 20% improvement in adherence to antihypertensive, lipid-lowering, and other cardiovascular medications correlated with reductions in cardiovascular events by 7%, 10%, and 9%, respectively; stroke by 17%, 13%, and 18%; and death by 12%, 9%, and 10%. The certainty of the evidence was moderate, suggesting that these effects are likely present. These findings emphasize the importance of enhancing medication adherence to improve clinical outcomes in CVD management.

**Conclusions:**

Evidence has demonstrated reductions in hard endpoints in both primary and secondary prevention through the control of conditions such as hypertension and elevated LDL cholesterol concentrations, as well as the benefits of antiplatelet therapy in atherosclerotic disease. However, additional studies are needed to better elucidate the relationship between adherence to cardiovascular medications and the improvement of critical clinical outcomes.

## Introduction

Cardiovascular diseases (CVD) remain a significant global health challenge and are the leading cause of mortality worldwide, accounting for an estimated 17.9 million deaths annually, or 31% of global deaths.^1^ Key risk factors such as hypertension, hypercholesterolemia, diabetes, smoking, obesity and sedentary lifestyle greatly contribute to the prevalence of CVD and the occurrence of premature deaths. The need to implement public health strategies focused on lifestyle modifications and preventive care is crucial.^2^

Economically, CVD impose a significant burden, not only due to direct healthcare expenses but also because of the indirect costs associated with lost productivity and long-term disability.^3^

The aim of this systematic review is, through the search for the best available scientific evidence, to evaluate the impact of adherence to cardiovascular medication treatments on clinical outcomes. This document will serve as a basis to the Brazilian Society of Cardiology Clinical Statement on the topic.

## Methods

The research question, framed in the PICO (patient/population, intervention, comparison and outcomes) format, was: in the adult population, aged 18 or over, what are the differences in clinical outcomes (death, stroke and myocardial infarction) between patients who adequately adhere or not to cardiovascular drug therapy? The protocol for this document was approved by the sponsor and is available for consultation upon request from the authors.

The rapid systematic review, which is the methodology employed in this document, belongs to the family of systematic reviews. It is a tool developed over the last decade aimed at maintaining methodological rigor while seeking the best possible evidence, but with modifications that expedite the execution time. Typically, these reviews inform medical societies or health-related institutions about the best available evidence based on a PICO-formatted question in a sensitive, transparent, and systematic manner. Leading institutions in the field of methodology have described the methods of this type of document.^4-6^

A comprehensive search was conducted across four databases: Medline, Embase, Lilacs, and the Cochrane Library, including all records from their inception until March 1, 2024.

Two researchers independently conducted the selection of studies and the quality assessment of the systematic reviews through an initial phase where both worked together until achieving a 90% agreement rate, after which each document could be evaluated by a single author. For data extraction, one researcher extracted all predefined variables onto a spreadsheet, while the second researcher independently extracted only the effect data. The quality assessment of the systematic reviews was conducted using two specific tools – AMSTAR 2 – A Measurement Tool to Assess Systematic Reviews and The Joanna Briggs Institute (JBI) Systematic Review Checklist.^7,8^ In addition, the GRADE framework was employed to evaluate the evidence quality and determine the strength of the recommendations where feasible.^5^

Articles selected for full reading were considered for inclusion in the study if they met the following criteria: (1) they were systematic reviews that included meta-analysis; (2) they reported risk estimates to assess the impact of adherence on major cardiovascular events (death from any cause, stroke, and myocardial infarction); (3) included patients aged 18 years or older; (4) evaluated at least one cardiovascular medication group such as antihypertensives, lipid-lowering, antithrombotic, or antiplatelet agents.

The following exclusion criteria were applied: (1) reviews that utilized fewer than two databases in their search; (2) reviews lacking detailed meta-analysis; (3) reviews that did not analyze the methodological quality of primary studies; (4) studies with low evidence quality as assessed by both tools (AMSTAR-2 and JBI). There were no language restrictions in the selection of studies.

Details of the search strategies and methodology employed in this rapid systematic review are provided in the supplementary material (Table 1S).

## Results

Of the 643 identified records identified, 15 were selected for full reading as they met the eligibility criteria. At the end, four systematic reviews with meta-analyses were included (Figure 1S, supplementary material). A list of excluded studies and reasons for exclusions are presented in the supplementary material (Table 2S).

All four systematic reviews included only observational studies, as expected. One review evaluated adherence to various types of cardiovascular medications and a broader range of outcomes,^9^ in addition to measuring the dose-response curve between adherence and complications. This review is considered the best available evidence and serves as the basis for the conclusions of this article. The other three reviews focused exclusively on one drug group, namely, antihypertensives,^10^ statins,^11^ or aspirin.^12^Table 1 presents the main characteristics evaluated in the included studies.

**Table 1:** – Characteristics of studies included in the rapid review

Author/ Year	Nº of studies included	Type of studies	Medication	Outcomes	Population	Adherence measure	Participants (n)	Median age	Female (%)	Funding
Liu/ 2021^9^	46	Observational	Statins, anti-hypertensive, antidiabetics, and antithrombotic agents	Cardiovascular events, stroke, and all-cause mortality	Hypertension / Dyslipidemia	MPR, PMC, CMA, PDC	4,051,338	60.1	44	No
Lee/ 2022^10^	161	Observational	Anti-hypertensive agents	Death and hospitalization	Patients with hypertension, >18 years; excluded: pregnant resistant hypertention	4-item or 8-item MMAS, pill counting, prescription refills, electronic pill boxes, biochemical assays, or electronic medication monitoring	27,785,595	57	57.1	No
Xu/ 2016^11^	6	Observational	Statin	All-cause mortality recurrence of CVD and revascularizetion	CVD	PDC	38,301	NR	NR	No
Biondi-Zoccai/ 2006^12^	6	Observational	Aspirin	Cardiovascular events	Patients at risk for or with CAD	NR	50,279	NR	NR	NR

CAD: coronary artery disease; CMA: cumulative medication adherence; CVD: cardiovascular disease; MMAS: Morisky Medication Adherence Scale; MPR: medication possession ratio; NR: not reported; PDC: Proportion of Days Covered; PMC: proportion of months covered by prescribed.

Source: Authors.

Liu et al.^9^ evaluated the association between vascular medication adherence and the risk of cardiovascular events, stroke, and all-cause mortality. The studies included patients in both primary and secondary prevention, involving healthy individuals, and individuals with hypertension, diabetes, dyslipidemia, and pre-existing CVD. The evaluated medications evaluated were lipid-lowering, antihypertensives, antidiabetics, and antithrombotic agents. The evaluated outcomes evaluated were death from all causes, stroke, and cardiovascular events (defined as any fatal or non-fatal coronary heart disease, myocardial infarction, heart failure, ischemic heart disease, or stroke or sudden cardiac death). Study-specific risk relative (RR) estimates were calculated per 20% increment of medication adherence and then pooled. Over four million patients distributed across 46 observational studies were included in the analysis, with quality assessment using the mean The Newcastle–Ottawa Scale score of 7.9 (maximum of 9), and an average follow-up period of 4.6 years. The analysis showed that increasing adherence to antihypertensive, lipid-lowering, and other cardiovascular medications by 20% reduced the risk of cardiovascular events by 7% [RR 0.93 (95% CI, 0.84-1.03), not significant], with the other findings demonstrating significance, 10% [RR 0.90 (0.88-0.92)], and 9% [RR 0.91 (0.84-0.98)], respectively. This increase in adherence also lowered the risk of stroke by 17% [RR 0.83 (0.78-0.89)], 13% [RR 0.90 (0.88-0.92)], and 18% [RR 0.91 (0.84-0.98)], and reduced all-cause mortality by 12% [RR 0.88 (0.82-0.94)], 9% [RR 0.91 (0.89-0.94)], and 10% [RR 0.89 (0.84-0.94)], respectively (Central Illustration). A sensitivity and subgroup analysis performed for the various outcomes did not show any significant difference in the pooled estimates associated with good adherence to cardiovascular medication.

Lee et al.^10^ conducted a systematic review and meta-analysis to estimate the global prevalence and consequences of nonadherence to antihypertensive medications among adult hypertensive patients. The analysis included several methods of measuring treatment adherence and involved 161 observational studies. This prevalence meta-analysis primarily aimed to assess blood pressure control, while also estimating secondary outcomes such as hypertension-related complications, all-cause hospitalization, and all-cause mortality. The results indicated that nonadherence to antihypertensive medications was associated with an increased odds ratio (OR) of death of 1.38 [95% CI, 1.35-1.41]. However, these results were based on only two studies with 1,653,763 patients and a median follow-up of 4.5 years. The analysis of certainty in the body of evidence, using the GRADE analysis, was deemed low for all outcomes. These results stem particularly from the observational nature of the studies.^5^

Xu et al.^11^ evaluated, through a meta-analysis, the relationship between adherence to the use of statins and long-term clinical consequences in patients with CVD (secondary prevention). The primary endpoint was all-cause mortality, measured by RR. The method of proportion of days covered (PDC) was used to quantify statin adherence (PDC ≥80% - good adherence and PDC <80% - poor adherence). A total of six studies were included in this meta-analysis, and the pooled RR favoring good statin adherence was 0.64 (indicating a 36% reduction in the risk of death from any cause) [95% CI, 0.52-0.80]. There was statistical significance, although the wide confidence interval indicated notable imprecision.

The last meta-analysis included was that of Biondi-Zoccai et al.^12^ in which the dangers inherent to aspirin withdrawal or non-compliance in subjects at risk for or with coronary artery disease were evaluated. Six studies were selected, and the result of the pooled estimate revealed that aspirin nonadherence/withdrawal was associated with a three-fold higher risk of major adverse cardiac events.

Table 2 provides the RR of the assessed outcomes, and the number of studies included in each summarized estimate.

**Table 2:** – Results of the meta-analysis of the included studies

Main author / Year	Medication group	All-cause mortality Measure (95% CI)	Nº of studies	Stroke Measure (95% CI)	Nº of studies	Cardiovascular events Measure (95% CI)	Nº of studies	Dose-response effect
Liu/ 2021^9^	Any cardiovascular medications*	RR 0.90 (0.87-0.92)	26	RR 0.84 (0.81-0.87)	23	RR 0.91 (0.88-0.94)	35	Yes
	Lipid-lowering agents*	RR 0.91 (0.89-0.94)	12	RR 0.87 (0.84-0.91)	7	RR 0.90 (0.88-0.92)	17	Yes
	Antihypertensives*	RR 0.88 (0.82-0.94)	8	RR 0.83 (0.78-0.89)	12	RR 0.93 (0.84-1.03)	13	Yes
	Others*^†^	RR 0.89 (0.84-0.94)	6	RR 0.82 (0.74-0.92)	4	RR 0.91 (0.84-0.98)	5	Yes
Lee/ 2022^10^	Antihypertensives	RR 0.75 (0.73-0.76) ^‡^	2	NR	-	NR	-	No
Xu/ 2016^11^	Statins	RR 0.64 (0.52-0.80)	6	NR	-	NR	-	No
Biondi-Zoccai/ 2006^12^	Aspirin	NR	-	NR	-	RR 0.37 (0.24-0.60) ^§^	6	No

(*) RR calculated for each 20% increase in medication adherence. (†) Others: antithrombotic and multiple medications. (‡) inverse relative risk, calculated from the original Odds Ratio result (1.38 [CI 1.35-1.41]) and a weighted probability of 8.01% of events over the follow-up period. (§) inverse relative risk, calculated from the original Odds Ratio result (3.14 [1.75-5.61]) and a weighted probability of 7.5% of events over the follow-up period. NR: not reported; OR: odds ratio; RR: relative risk; Rr: risk ratio. The statistical significance level adopted across all studies was 5%.

Source: Authors.

Results of the AMSTAR-2 analysis and the JBI critical appraisal checklist are presented in Tables 3S and 4S (supplementary material). Despite the differing results between these two tools, the authors deemed the overall quality of the four included systematic reviews to be satisfactory.

The authors performed a separate GRADE assessment for the study by Liu et al.,^9^ based on the risk of bias and the characteristics extracted from the study. This evaluation addressed all the drug groups and the various outcomes. In nearly all instances, the evidence achieved a moderate level of certainty, largely due to the presence of a dose-response gradient. However, the certainty of the evidence for cardiovascular event outcomes associated with hypertensive medications was deemed very low. This assessment was based on the imprecision of the RR estimates, which intersected the null effect line. Consequently, it was recommended not to increase the certainty score for these specific outcomes.

The evidence summary table is presented in the supplementary material (Table 5S).

## Discussion

Medication adherence is a significant health issue today, and the Brazilian Society of Cardiology has taken a pioneering approach by addressing this topic, seeking the best scientific evidence to support their position statement.

Answering the questions posed for this review is not a simple task. It was anticipated that there would be no clinical trials on this topic, as it is not feasible to randomize patients to adhere or not adhere to cardiovascular medications with proven benefits. Therefore, it was necessary to use systematic reviews of observational studies, which start with a low degree of certainty of evidence but attempt to aggregate the results of multiple studies in order to improve confidence in the effect estimate.

Additionally, it was expected that these studies on adherence would exhibit significant heterogeneity for multiple reasons: the wide variety of methods for measuring adherence, the different groups of cardiovascular medications, the diverse populations (primary vs. secondary prevention, adults vs. elderly, etc.), the plurality of clinical outcomes, and the varying quality of the studies, among other possibilities.

Medication adherence is often quantified using various cutoff points and categorized into distinct levels, providing essential insights into patient behavior and treatment efficacy. Adherence is typically categorized based on percentage thresholds reflecting the proportion of prescribed doses a patient takes over a specific period. Commonly, “high adherence” is defined as taking 80% or more of the prescribed doses, “medium adherence” as taking 50-79%, and “low adherence” as taking less than 50%. These thresholds are extensively utilized in clinical research to assess intervention effectiveness and are crucial for understanding their impact on clinical outcomes.^13^ The reference study by Liu et al.^9^ evaluated the impact of a 20% increase in medication adherence (for instance, from 80% – the most used cutoff in the literature to distinguish good adherence – to 100%). The study demonstrated a significant decrease in major cardiovascular outcomes when the cardiovascular drugs were used as prescribed.

A meta-analysis examining statin therapy revealed that adherence rates at one year of follow-up differed significantly between study designs: 49.0% in observational studies compared to 90.3% in randomized controlled trials. This discrepancy suggests that adherence in randomized clinical trials, where there is greater control, may be overestimated compared to reality.^14^

These adherence cutoffs are employed across various medication types, including both chronic and acute therapies, to standardize research methodologies and enable meaningful comparisons. For example, the World Health Organization indicates that adherence levels above 80% are generally required to achieve optimal therapeutic outcomes in most chronic conditions.^15^ However, certain conditions demand more specific adherence rates; for instance, antiretroviral therapy for HIV may require adherence levels as high as 95% to effectively suppress viral loads.^16^ While these thresholds are pivotal for research uniformity, their applicability can vary based on the therapeutic window of the medication, the specific health condition, and individual patient factors. This variability underscores the need for adherence strategies that are tailored to maximize patient outcomes, suggesting a more nuanced application of these metrics depending on the therapeutic requirements and patient circumstances.^16^

Establishing the multifaceted barriers to adherence is essential for optimizing cardiovascular outcomes. Factors such as patient-related issues (e.g., forgetfulness, beliefs about medication, perceived side effects), socioeconomic challenges (e.g., medication cost, patient education), and healthcare system obstacles (e.g., complex medication regimens, lack of follow-up) must be systematically addressed to enhance adherence rates.^15,17^ Different models and theories attempt to address the complexities underlying these behaviors. For example, the Health Belief Model suggests that patient perceptions of the severity of their condition, the potential benefits of treatment, and barriers to care can influence adherence and persistence.^18^ Furthermore, the World Health Organization identifies some factors that impact adherence, categorizing them into patient-related, condition-related, therapy-related, socioeconomic, and healthcare system factors.^15^ Patient education, simplified drug regimens, and improved healthcare provider-patient communication are essential for enhancing adherence and ultimately improving clinical outcomes in CVD management.^17^

One aspect warranting also consideration in medication adherence studies is dosage intensity. For example, one investigation evaluated adherence across different statin intensity levels — low, moderate, and high. The findings indicated that adherence rates at 12 months were 57.2% for low-intensity statins, 46.5% for moderate-intensity statins, and 37.9% for high-intensity statins, with adherence defined as achieving at least 80% medication compliance.^19^ A further study identified a statistically significant disparity in adherence between low and high-dosage statins, suggesting that regimes involving high-intensity statins are linked to reduced adherence compared to those involving lower-intensity statins.^20^ This observation appears to contradict a more recent article, which posits that following the introduction of new American College of Cardiology/American Heart Association (ACC/AHA) guidelines, a higher proportion of patients with atherosclerotic CVD are not only prescribed high-intensity statins but are also more likely to adhere to their treatment regimen.^21^

Another aspect on this subject is the use of fixed-dose combination (FDC) therapy, which has emerged as a promising strategy to enhance medication adherence in patients with CVD. FDC therapy simplifies the treatment regimen by combining multiple medications into a single pill, which can reduce pill burden and improve patient compliance. Studies have demonstrated that FDC therapy is associated with higher adherence rates and better clinical surrogate outcomes compared to multiple-pill regimens. For instance, a study found that patients on FDC therapy had a 24% higher adherence rate and significantly improved blood pressure control.^22^ Similarly, a meta-analysis reported that FDC therapy led to a 26% reduction in the risk of nonadherence and better management of cardiovascular risk factors.^23^ These findings indicate the potential benefits of FDC therapy in optimizing cardiovascular treatment and enhancing patient adherence.

This review is not free of limitations. Firstly, only systematic reviews that included observational studies were selected. Observational studies, by their nature, have a greater margin of imprecision, primarily due to unmeasured or even unknown confounding factors. This justifies the requirement for primary studies to identify and adjust for confounding factors deemed important, and for systematic reviews to assess the quality of the primary studies, ideally including only high-quality research. Because some of the selected documents did not meet these criteria, they were excluded from our review.

Secondly, given the Brazilian Society of Cardiology’s interest in various classes of cardiovascular medications and critical clinical outcomes, as well as the significant variability in adherence measurement methods, substantial heterogeneity among the studies was anticipated. In reference to the document by Liu et al.,^9^ it was decided not to penalize the study for heterogeneity, despite the Cochran Q and I^2^ statistics indicating significant variability across studies. It presented similar effect estimates with consistent direction when analyzing the different medication groups by outcome. Furthermore, the confidence intervals demonstrated overlap, and several subgroup analyses did not alter the overall results, nor did the sensitivity analysis. Despite the heterogeneity being only partially explained, the potential benefits of good adherence to effective cardiovascular medications are substantial and should not be underestimated. The decision not to penalize the GRADE assessment in relation to this characteristic may not be unanimous.

Finally, regarding the assessment of the certainty of evidence using the GRADE tool, ideally, it should be conducted by the authors in each systematic review with meta-analysis. However, of the four studies included, only one presented such an analysis. In the reference study, this assessment was conducted based solely on the extracted information, without access to the primary sources.

While robust evidence supports reductions in hard endpoints in both primary and secondary prevention through the control of clinical variables such as blood pressure^24,25^ and LDL-cholesterol concentrations,^26,27^ as well as the benefits of antiplatelet therapy in atherosclerotic disease,^28^ there remains a scarcity of studies demonstrating correlations between adherence to cardiovascular medications and attenuation of critical clinical outcomes.

Despite the limitations inherent to observational studies, the evidence of the risks of poor medication coverage reinforces the global need to implement strategies that improve adherence to cardiovascular treatments.

## Conclusion

This systematic review demonstrates the significant impact of good adherence to cardiovascular medication treatment on clinical outcomes.

According to the GRADE methodology, there is moderate certainty of evidence that patients who adhere to their prescribed cardiovascular medications experience a reduction in death, stroke, and cardiovascular events compared to individuals with lower adherence.
